# Development and Validation of HPLC-DAD Method with Pre-Column PMP Derivatization for Monomeric Profile Analysis of Polysaccharides from Agro-Industrial Wastes

**DOI:** 10.3390/polym14030544

**Published:** 2022-01-28

**Authors:** Aleksandra Vojvodić Cebin, Draženka Komes, Marie-Christine Ralet

**Affiliations:** 1 Department of Food Engineering, Faculty of Food Technology and Biotechnology, University of Zagreb, Pierottijeva 6, 10 000 Zagreb, Croatia; 2 INRAE, UR BIA Biopolymers-Interactions-Assemblies, F-44316 Nantes, France; marie.ralet@inrae.fr

**Keywords:** agro-industrial waste, HPLC, method validation, PMP derivatization, polysaccharides, sugar profiling

## Abstract

The instrumental analysis of complex mixtures of sugars often requires derivatization to enhance the method’s selectivity and sensitivity. 1-Phenyl-3-methyl-5-pyrazolone (PMP) is a common sugar derivatization agent used in high-performance liquid chromatography (HPLC). Although many C18 column applications for PMP–sugar derivative analysis have been developed, their transferability is not straightforward due to variations in column chemistry and preparation technology. The aim of this study was to develop and validate an application for Zorbax Extend C18 columns for the analysis of 8 neutral and 2 acidic sugars commonly found in plant polysaccharides. The method was further compared to well-established alditol acetates and *m*-hydroxydiphenyl methods and employed for sugar profiling of selected agro-industrial wastes. The most influential separation factors were the mobile-phase pH and acetonitrile content, optimized at 8.0 and a 12–17% gradient, respectively. The method showed excellent linearity, repeatability and intermediate precision. High sensitivity was achieved, especially for neutral sugars, with an accuracy error range of 5–10% relative standard deviation. The sugar profiling results were highly correlated to the reference method for neutral sugars. The HPLC method was highly applicable for the evaluation of polysaccharides in selected wastes and showed advantages in terms of simplicity, accuracy in acidic sugar determination and suitability for their simultaneous analysis with neutral sugars.

## 1. Introduction

Carbohydrates comprise a very large group of biomolecules that are categorized into monosaccharides, oligosaccharides and polysaccharides. Polysaccharides are high-molecular weight carbohydrates that consist of monosaccharide units linked by glycosidic linkages. It is estimated that more than 90% of the carbohydrate mass in nature is in the form of polysaccharides [1]. As such, polysaccharides are highly represented as native and industrial food components, exhibiting nutritive, technological, sensorial and functional or bioactive properties [1,2]. Due to the high number of monosaccharides and the ways they can be connected, the variety of polysaccharides is extremely large. The application of polysaccharides in food and other industries relies on their specific properties, which are reflected in their monomeric composition and organization. Thus, accurate determination of the monomeric composition is a crucial step in polysaccharide evaluation in order to elucidate properties and structure, as well as to understand the structure–activity relationship [3]. The analysis of monosaccharides in complex mixtures is challenging due to several reasons, including the (i) incomplete hydrolysis of polysaccharides, (ii) the formation of degradation products during hydrolysis, (iii) the similarity of the structures between monosaccharides, (iv) the hydrophilic character and (v) the lack of chromophores or fluorophores in the molecules [3,4].

The application of liquid chromatography in carbohydrate analysis started as paper and thin-layer chromatography in the 1960s, followed by high-performance liquid chromatography (HPLC) in the 1980s [5]. To date, along with the strong development of the instrumental analysis methodology, HPLC has remained a preferred choice for carbohydrate analysis in general [6,7]. Among liquid chromatography techniques, high-performance anion-exchange chromatography coupled with pulsed amperometric detection (HPAEC-PAD) has become the main method for direct analysis of the monomeric composition of polysaccharides due to the ability to analyze neutral and acidic sugars simultaneously with high sensitivity [3,8]. However, the absorption of CO_2_ and the formation of carbonate in the mobile phase can result in loss of the column capacity, leading to the co-elution of monosaccharides [3]. In addition, peak overlapping between xylose and mannose, as well as between rhamnose and arabinose, is one of the problems with this technique [9]. 

Despite the progress made in the direct chromatographic analysis of sugars, various derivatization protocols are often employed to meet specific analysis demands. Derivatization in sugar analysis allows the enhancement of chromatographic separation due to the changes in the physicochemical properties of the derivatives, which is especially important for complex mixtures of sugars, as well as allowing for higher method sensitivity by enabling the use of more sensitive modes of detection, such as fluorometry and UV spectroscopy [10]. Many pre-column derivatization procedures have been developed for HPLC and GC analyses [4,10,11]. The selection of the derivatization procedure is usually based on the size of sugar molecules, their solubility in solvents used in the derivatization and the desired derivative properties for the selected mode of chromatographic separation or detection, generally in order to achieve high reaction yields and to form high-purity derivatives, ensuring their stability and lowering the costs of the method [10].

The derivatization procedure with 1-phenyl-3-methyl-5-pyrazolone (PMP) is frequently used in HPLC sugar analysis [12]. The procedure was originally developed by Honda et al. [13], employing mild reaction conditions. The reaction is proceeded on the sugar-reducing end, where in a stepwise mechanism two PMP molecules react with one aldehyde group to form a bis-PMP–sugar derivative. The reaction requires an alkaline medium to form reactive PMP enolate ions [5,12] and a high excess of PMP reagent [14]. As the aldehyde group is required for reaction, alcohol sugars cannot be analyzed as PMP derivatives [12,15]. Non-reducing sugars such as sucrose and cyclodextrins do not react with PMP [12]. In addition, fructose, although a reducing sugar, cannot form PMP derivatives, probably due to the lower reactivity of the keto group and to the steric hindrance caused by molecule configuration. In the original procedure, the optimized conditions comprised sodium hydroxide (NaOH) at 0.3 M concentration, PMP methanolic solution (0.5 M), a reaction temperature of 70 °C and a reaction time of 30 min [13]. In more recent attempts to optimize the reaction, the reaction time and temperature were reconsidered [14,15,16], along with the base catalyst concentration and amount of PMP [14]. It was found that the presence of different functional groups (e.g., carbonyl-, amino-) and their positions in the sugar molecules result in differences in the reaction times needed to obtain maximal yields of a respective PMP derivative [15].

The physicochemical properties of PMP–sugar derivatives reflect those of PMP molecules, so in general they exhibit a hydrophobic character due to the presence of a pyrazolone ring enhanced by the presence of phenyl- and methyl-groups. Additionally, the PMP–sugar molecule has the properties of a weak acid, being negatively charged at higher pH values due to the keto-enol tautomerization of the pyrazolone ring [5]. Therefore, different types of C18 columns are used in HPLC, whereas the mobile phase is buffered at different pH levels, usually at neutral to weakly basic values (6.7–8.0) [16,17,18], but also at acidic pH levels (approximately 4.5–5.5) [14,15,19]. From an analytical standpoint, probably the most important characteristic of PMP–sugar derivatives is their strong absorption in the UV region (245 nm, ε = 29 400 L/mol cm for PMP–glucose in ethanol at 245 nm [13]). MS detection is also applicable [14,19]. However, in the derivatization protocol, a fair amount of sodium chloride is generated, so some analyses may require desalting or the use of a different base catalyst. PMP derivatization was successfully applied in various studies employing carbohydrate analysis to determine monosaccharides and oligosaccharides qualitatively and quantitatively, as well as to determine the monomeric compositions of plant and fungal polysaccharides [12,20].

With the rising interest in the reutilization of agro-industrial wastes, especially of lignocellulose, monomeric profiling of plant polysaccharides has gained additional importance, while simple, effective and relatively cheap methods are highly needed for further progress in the field. Solid plant agro-industrial wastes comprise agricultural residues and food processing by-products generated in the field and in the factory. These materials are abundant in cell wall polysaccharides and can be recovered and reused directly or modified or subjected to bioconversion and fermentation to produce added-value products [21]. One of the most interesting reutilization strategies for plant waste polysaccharides is within the food industry, where they can be used conventionally or more importantly as functional ingredients in the development of innovative food products. For such purpose, the reutilization of pectin from pectin-rich waste products [22] and lignocellulose in the production of xylooligosaccharides [23] and xylitol [24] is being extensively studied.

The determination of the total monomeric sugar profile of agro-industrial wastes is usually a primary step in the evaluation of their potential in terms of carbohydrate reusability, for example for the extraction of different polysaccharides or for sugar bioconversion or fermentation to other products. Monomeric profiling also enables the characterization of extracted polysaccharides and the evaluation of their recovery rates. PMP derivatization has been successfully employed to evaluate carbohydrates in various types of agro-industrial wastes, such as citrus peel [25], bamboo shoots [26], red algae [27] and spent mushroom [28], as well as to analyze xylooligosaccharides derived from lignocellulosic biomass [18,29]. However, regarding the high number of available C18 columns from different manufacturers, which differ in column chemistry and preparation technology, combined with the impacts of pH on the properties of PMP–sugar derivatives, the developed HPLC methods for the analysis of PMP–sugar derivatives are not easy to transfer. In line with the need for further developments for carbohydrate analysis in general, there is great benefit in reporting validated application references. Moreover, even though the PMP derivatization approach has been in use for a long time, comparative studies on the applicability of different detectors, columns or other reference methods are still lacking in the literature [30].

Therefore, the aim of this paper was to develop and validate an HPLC-DAD method with pre-column PMP derivatization for simultaneous analysis of 8 neutral and 2 acidic sugars and to further evaluate its applicability for analyzing cell wall polysaccharides from selected agro-industrial (mainly lignocellulosic) wastes, while comparing it to the well-established GC-FID (alditol acetates) and colorimetric (*m*-hydroxydiphenyl) methods. The results of this study will serve as a much-needed analytical case study, as well as a compositional reference in the fast-growing field of reutilization of polysaccharides from agro-industrial wastes.

## 2. Materials and Methods

### 2.1. Samples

In total, 5 agro-industrial wastes were used, namely sugar beet pulp (SBP), walnut shell (WS), cocoa bean husk (CBH), onion peel (OP) and pea pod (PP). SBP was supplied as a byproduct from sucrose production (Viro Sugar Factory, Virovitica, Croatia), in dried, pelleted form. CBH was supplied as a byproduct from chocolate production (Zvečevo Confectionery Industry, Požega, Croatia), collected after cocoa bean roasting and delivered in originally dried form. WS and PP were prepared for domestic use, i.e., by deshelling (WS) and dehulling (PP), while OP was collected as a restaurant waste and consisted of dry orange-brown skin, 1–2 outer fleshy leaves, tops and roots and degraded portions of onion bulbs. OP and PP were additionally dried in a laboratory dryer (ST-06; Instrumentaria, Zagreb, Croatia) at 50 °C for approximately 48 h or until reaching approximately 10% moisture. Samples were repeatedly homogenized in a coffee mill for domestic use until a fine powder was produced. Due to hardness, WS powder was prepared in a laboratory ball homogenizer (Mixer Mill MM 400; Retsch, Haan, Germany). Powdered samples were sieved through a 450 µm screen and approximately 500 g of each sample was collected. Samples were stored in paper bags in a cool and dry place, along with a small portion of silica gel to prevent moisture accumulation.

### 2.2. Chemicals

All chemicals used were of analytical or HPLC/GC grade. PMP reagent was obtained from Acros Organics (Geel, Belgium). The sugar standards D-(+)-mannose, D-(+)-galactose, D-(-)-xylose, D-(+)-arabinose and L-(+)-rhamnose monohydrate) were obtained from LGC Standards (Teddington, UK). D-8+)-glucose monohydrate was obtained from Fluka (Buch, Switzerland). L-(-)-fucose was obtained from Acros Organics (Geel, Belgium). D-(+)-galacturonic acid, D-glucuronic acid and D-(-)-ribose were obtained from Sigma-Aldrich/Merck (USA). 

### 2.3. Preparation of Alcohol-Insoluble Residues

Alcohol-insoluble residues (AIR) of selected agro-industrial wastes were prepared via 3 successive extractions in 70% (*v*/*v*) ethanol. Samples (10 g) were mixed with the solvent at a 1:10 (*w*/*v*) ratio and stirred for 30 min on a magnetic stirrer (RT 5; IKA, Staufen, Germany) at room temperature. The residues were recovered by vacuum filtration (Whatman 4) after each extraction and finally dried by solvent exchange (2 ethanol (96%) washings and 1 acetone washing, both at 1:2 (*w*/*v*) ratio).

### 2.4. Complete Acid Hydrolysis

Complete acid hydrolysis of AIRs was conducted prior to derivatization [31,32]. Approximately 25 mg of AIR was carefully weighed in a glass screw-cap tube, 0.25 mL of 72% (*v*/*v*) sulfuric acid was added and distributed through the sample using a glass rod, then the sample was left to stand for 30 min at room temperature with occasional stirring with the glass rod. Next, 2.25 mL of water and 0.5 mL of previously prepared internal standard (5 mg/mL *myo*-inositol or 2.4 mg/mL ribose) were added and the capped tubes were placed in a thermoblock heater at 100 °C for 2 h. Once cooled, the hydrolysates were centrifuged (SL 8R with fixed angle rotor; Thermo Fisher Scientific, Waltham, MA USA) at maximum speed for 10 min at room temperature. An aliquot of 1 mL of supernatant was taken for analysis. Along with the samples, a mixture of sugar standards at 1 mg/mL each was subjected to hydrolysis at 100 °C in 1 M sulfuric acid for 2 h and was set as a reference sample to account for losses during hydrolysis. Complete acid hydrolysis was performed in triplicate.

### 2.5. Determination of Monomeric Composition 

#### 2.5.1. Determination of Neutral Sugars by GC-FID

This method is based on the reduction and acetylation of sugar molecules to alditol acetates, followed by GC-FID analysis [32,33]. *Myo*-inositol was used as an internal standard. An aliquot of 0.5 mL of acidic hydrolysate (see Section 2.4) was alkalized via the addition of 0.15 mL of ammonia solution (25%) in a glass screw-cap tube and checked for pH (approximately 9). Then, 0.1 mL of freshly prepared 100 mg/mL sodium borohydride solution in 3 M ammonia was added, then the tubes capped and incubated at 40 °C for 1 h. Once cooled in an ice bath, the excess of NaBH_4_ was degraded via the addition of 2 portions of 0.05 mL of glacial acetic acid. An aliquot of 0.15 mL was acetylated via the addition of 2 mL acetic anhydride and 0.2 mL N-methylimidazole while allowed to stand for 20 min at room temperature. The reaction was stopped by the addition of 5 mL of water and vigorous vertexing. The prepared alditol acetates were extracted in 1.5 mL of dichloromethane and subsequently washed with 2 portions of water (5 mL).

The GC-FID analysis was performed using an Autosystem XL GC (Perkin Elmer, Boston, MA, USA) with a DB225 30 m × 0.32 mm, 0.25 µm column (SGE Analytical Sciences, Australia). The injection volume was 1 µL in split mode (1:50) and the injector temperature was set to 220 °C. Hydrogen was used as the mobile phase at a 4 mL/min flow rate (Hydrogen Generator 9400; Packard Instrument Company, Meriden, CT, USA). The oven temperature was 205 °C and the analysis was conducted isothermally for 15 min. The detector was set at 220 °C and −200 V voltage. Chromatograms were collected and analyzed using TotalChrom software (Thermo Fisher Scientific, Waltham, MA, USA). The identification of peaks was performed by comparison of the relative retention times of samples and standard mixture chromatograms. The results were calculated using the internal standard concentration and response factors as a mass percentage (% dry matter basis (dmb)) of each monomer, i.e., their anhydro forms using polymerization factors of 0.9 for hexoses, 0.88 for pentoses and 0.89 for deoxy sugars. 

#### 2.5.2. Colorimetric Determination of Uronic Acids

The method was performed following the procedures used by Blumenkrantz and Asboe-Hansen [34], Ahmed and Labavitch [35], Thibault [36] and Filisetti-Cozzi and Carpita [37]. The method is based on the formation of pink-red products from the reaction of furfuric derivatives of sugars in the presence of strong acids at elevated temperature and *m*-hydroxydiphenyl. The analysis was performed in a Sanplus System semi-automated analyzer (Skalar Analytical, Breda, the Netherlands) according to a verified laboratory protocol. The reagents were: (i) concentrated sulfuric acid, (ii) sodium tetraborate decahydrate solution (0.0125 M) in concentrated sulfuric acid and (iii) *m*-hydroxydiphenyl (400 mg/L solution in 0.125 M sodium hydroxide solution), with (i), (ii) and (iii) being combined to determine total uronic acids, while (i) and (iii) were combined for galacturonic acid. The signals were recorded using a photometer (520 nm) and correlated to the height of outgoing peaks on the detector to represent each sample. The system was calibrated for galacturonic acid and additionally for glucuronic acid for total uronic acid determination at 10–100 µg/mL over 6 points. To account for neutral sugar interferences, glucose and xylose were analyzed at 5 rising points, covering the concentration range as determined by GC-FID analysis (see Section 2.5.1). The results were calculated as mass percentages (% dry matter basis (dmb)) of the respective uronic acids, i.e., their anhydro forms using polymerization factors of 0.907.

#### 2.5.3. Simultaneous Determination of Neutral and Acidic Sugars by HPLC-DAD

For HPLC analysis, ribose was used as an internal standard (being added to the sample before complete acid hydrolysis, see Section 2.4).

##### Derivatization Using 3-Methyl-1-Phenyl-2-Pyrazoline-5-One

Prior to derivatization, aliquots of 1 mL of acidic hydrolysates (see Section 2.4) were neutralized (pH 6.5–7.0) via the gradual addition of an equimolar (to the concentration of H_2_SO_4_) quantity of solid calcium carbonate. The obtained slurry was briefly centrifuged, and the clear supernatant was collected for derivatization. Derivatization was performed following the procedure used by Sun et al. [38], with modification of the reaction time (60 min). 

##### Optimization of HPLC Mobile-Phase Composition

An Agilent 1200 Series (Agilent Technologies, Santa Clara, CA, USA) chromatographic system was used for all HPLC analyses, consisting of a vacuum degasser, quaternary pump, autosampler with thermostat, column compartment thermostat and photodiode array detector (DAD). The system was coupled with a Zorbax Extend C18 (4.6 mm × 250 mm; 5 µm) column (Agilent Technologies, Santa Clara, CA, USA). The mobile phase involved two components, combining (A) 20 mM phosphate buffer of varying pH levels and (B) acetonitrile (AcN) in isocratic methods. The selected pH values of the buffer component (A) were 7.0, 7.5 and 8.0, while the contents of AcN (B) were 15, 16, 17 and 18% (vol.). The flow rate was set at 1 mL/min, the column temperature at 30 °C, the injection volume at 10 µL and the detection at λ = 245 nm (DAD). The duration of all established isocratic methods was 80 min in order to assure that all of the peaks had been eluted.

##### HPLC-DAD Analysis under Optimized Conditions

The optimized conditions for HPLC-DAD analysis comprised a two-component mobile phase consisting of (A) 100 mM sodium phosphate buffer at pH 8.0 and (B) acetonitrile. The elution was performed in gradient as follows: 0 min–12% B, 35 min–17% B, 36 min–20% B, 45 min–20% B, 46 min–12% B, 65 min–12% B. The other parameters were as stated in the previous paragraph, except for the column temperature, which was 25 °C, and run time. Chromatograms were collected and analyzed using ChemStation software (Agilent Technologies, Santa Clara, CA, USA). Identification of the peaks was performed by comparison of relative retention times of samples and standard mixture chromatograms. The results were calculated using internal standard concentrations and response factors as mass percentages (% dry matter basis (dmb)) of each monomer, i.e., their anhydro forms, using polymerization factors of 0.9 for hexoses, 0.88 for pentoses, 0.89 for deoxy sugars and 0.907 for uronic acids.

##### Validation of Optimized HPLC-DAD Method

The optimized HPLC-DAD method was validated for linearity, limits of detection and quantification, precision and recovery, as described elsewhere [16,17].

Linearity was established for a concentration range of 10–400 µg/mL, distributed over 10 points for each monomer, while the internal standard concentration was the same for each point (400 µg/mL). The sample (standards mixture) was prepared in duplicate. 

The limit of detection (LOD) and limit of quantification (LOQ) were determined using Equations (1) and (2), respectively.

LOD = 3.3·(σ/S)(1)

LOQ = 10·(σ/S)(2)

where σ represents the residual standard deviation of the regression line and S represents the slope of the regression line. Both were calculated using the LINEST function (Excel, MS Office 2013). Data were collected from 7 points in duplicate, covering a range of 2.5–100 µg/mL.

The precision of the retention time and area were evaluated as repeatability (interday precision) and intermediate precision (intraday precision) via sequential (*n* = 5/day) analysis of a mixture of standards (250 µg/mL each) prepared in triplicate for three days. Each day, the mobile phase was changed and prepared by a different analyst. The results are presented as percentages of the relative standard deviation (%RSD), calculated using Equation (3).

%RSD = SD/AVG·100(3)

where SD represents the standard deviation and AVG represents the average of a defined number of repetitions per evaluated parameter. 

The recovery evaluation comprised spiking of a known sample (acidic hydrolysate of cocoa bean husk (CBH) AIR) at 3 different levels, namely 25/100 µg/mL, 50/200 µg/mL and 75/300 µg/mL, where the higher concentration at each level was used for the two most represented monomers in the sample, while the lower was used for the rest. The results were calculated as a percentage (%) from the expected value and %RSD of 3 parallel sample preparations.

### 2.6. Statistical Analysis

The analysis was used to determine the statistically significant differences between HPLC and GC/MHDP results for the contents of sugar monomers. It was performed as a one-way ANOVA (SPSS 17.0; IBM, Armonk, NY, USA) at the significance level of α = 0.05 with Tukey’s post-hoc test.

## 3. Results and Discussion

In this study, we developed and validated an HPLC-DAD method with pre-column PMP derivatization for the determination of the monomeric profiles of polysaccharides from various plant-derived agro-industrial wastes. The derivatization protocol is simple to follow and involves low-cost reagents, relatively mild conditions and standard laboratory equipment. The analytical method involves a C18 column coupled with a DAD detector, both of which have diverse and broad analytical uses, and the column is relatively cheap in comparison to specialized columns used for carbohydrate analysis.

### 3.1. Influence of the Mobile Phase Composition

**Figure 1:**
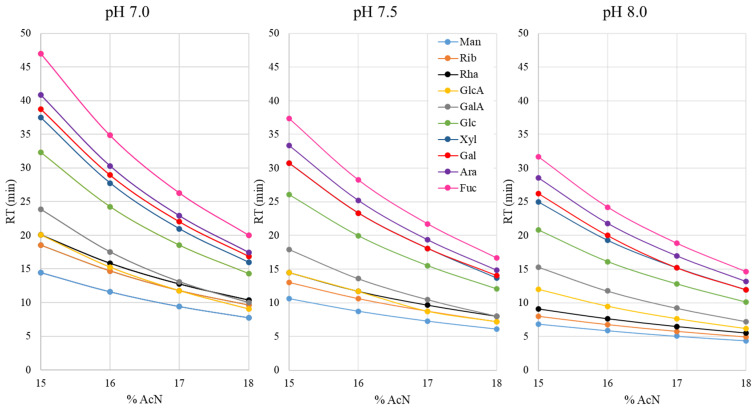
The effects of the pH value of the buffer component of the mobile phase and the organic component content (acetonitrile (AcN)) on the retention times of analyzed PMP-derivatized monomeric sugar standards (Man—mannose; Rib—ribose; Rha—rhamnose; GlcA—glucuronic acid; GalA—galacturonic acid; Glc—glucose; Xyl—xylose; Gal—galactose; Ara—arabinose; Fuc—fucose).

Initially, we tried to adopt the previously reported analytical methods of analysis for PMP–sugar derivatives. It was shown that the retention time and separation efficiency were not easy to reproduce by applying the already established conditions to a C18 column that was at our disposal. This was most probably due to the slight changes in the chemistry and preparation technology for the same type (C18) of HPLC columns used in different studies. Therefore, we started to develop a new application for the Zorbax Extend C18 (Agilent Technologies) column. In the first step, we aimed to define the impacts of the pH value of the buffer component of the mobile phase and the content of the organic component (acetonitrile). A set of 12 isocratic methods, combining 3 pH values of the buffer (7.0, 7.5 and 8.0) and 4 acetonitrile contents (15, 16, 17 and 18% (vol.)), was employed for the analysis of the standard mixture. The obtained chromatograms were evaluated for the shift in retention time for each monomeric standard (Figure 1). 

**Figure 2:**
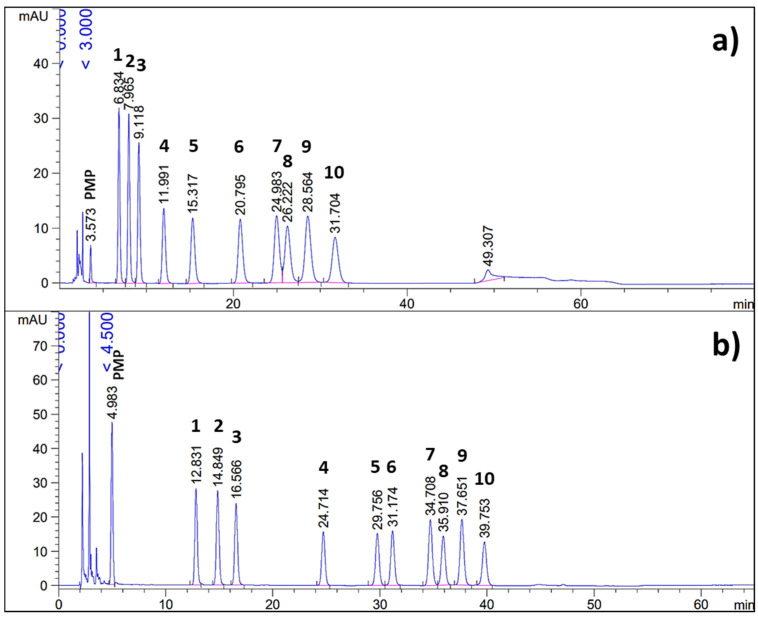
Chromatograms of 10 monomeric PMP–sugar derivatives: (**a**) analyzed by isocratic elution and mobile phase combining the buffer component at pH 8.0 and 15% acetonitrile (AcN) as the organic component; (**b**) analyzed by gradient elution and mobile phase combining the buffer component at pH 8.0 and AcN at 12–17%; 1-mannose, 2-ribose, 3-rhamnose, 4-glucuronic acid, 5-galacturonic acid, 6-glucose, 7-xylose, 8-galactose, 9-arabinose, 10-fucose (all PMP derivatives).

In this study, we aimed to optimize the separation of 8 neutral sugars (mannose, ribose, rhamnose, glucose, xylose, galactose, arabinose and fucose) and 2 acidic sugars (glucuronic and galacturonic acid) commonly found in plant polysaccharides. The derivatization protocol and the analytical method were found to be suitable for simultaneous analysis of neutral and acidic sugars. As can be seen from Figure 1, the pH and acetonitirle content (%AcN) limits were well-chosen for this type of C18 column, since lower values would result in long run times and more pronounced peak broadening, while higher values would result in impaired resolution. The increase in pH as well as in %AcN decreased the retention times of all monomeric sugars, in agreement with previously published data [16]. The retention of uronic acids seemed to be the most affected by the mobile-phase composition, resulting in changes in the elution order or coelution with ribose and rhamnose. This was pronounced at pH values of 7.0 and 7.5, while at pH 8.0 the chromatographic migration of uronic acids with the increasing AcN content was much more consistent as compared to other monomers and the elution order was constant. Among neutral sugars, the pH and AcN content had the greatest impacts on the separation of xylose and galactose, which was unaffected only at pH 7.0 over the whole %AcN range, while at pH 7.5 the monomers were coeluting. At pH 8.0, their separation was notably affected above 16% of AcN in the mobile phase, resulting in complete coelution. To the best of our knowledge, the mechanism behind the separation of PMP–sugar derivatives on C18 columns is still not completely resolved. From the results, however, it can be observed that the PMP–sugar derivatives are ionizable molecules. Their ionization derives from keto-enol tautomerization, i.e., the dissociation of the enol group in the pyrazolone ring of the PMP molecule at higher pH values. The tautomerization and the ionization are affected by the pH [5]. Ionized molecules are less retained on C18 columns than non-ionized ones. From the 12 tested mobile phases in isocratic runs, the combination of pH 8.0 of the buffer component and 15% of AcN as the organic component resulted in the best separation of all tested monomers (Figure 2a). This served as the basis for establishing the gradient elution process in order to compromise between further enhancement in the separation of xylose and galactose and elution time, as well as to minimize peak broadening. The gradient was established using 12–17% of AcN, while the pH value of the buffer component remained at 8.0. It was shown that the ionic strength of the buffer also had an impact on the chromatographic migration of PMP–sugar derivatives and it was set at 100 mM for optimal separation. Under the described conditions, all analyzed sugars were separated within approximately 40 min of analysis (Figure 2b).

### 3.2. Validation of the Developed HPLC-DAD Method

The developed HPLC method was validated in terms of linearity, limits of detection and quantification, precision and recovery. Parameters of the linearity and limits of detection and quantification are presented in Table 1.

A highly linear response was established for all PMP–sugar derivatives in the concentration range of 10–400 µg/mL. The limits of detection ranged between 1.17 µg/mL (rhamnose) and 4.83 µg/mL (fucose), while the limits of quantification ranged between 3.55 µg/mL (rhamnose) and 18.32 µg/mL (glucuronic acid). It was observed that the LOD and LOQ increased for the later-eluting sugars. This can possibly be explained to some extent by the peak broadening effect at the end of the analysis, resulting from the relatively long run time and narrow AcN gradient in combination with the relatively low starting %AcN in the mobile phase.

The precision (repeatability and intermediate precision) parameters are presented in Table 2. Repeatability was determined by extracting minima and maxima of 9 average values (3 consecutive days and 3 replicate samples), each obtained from 5 replicate analyses (5 injections daily for each sample), for the retention time (RT) and area (A).

The min–max %RSD values are presented for the absolute retention time and area, as well as in relation to ribose, which was used as the internal standard. The minimum and maximum intermediate precision values were taken from 3 average values (3 samples) obtained from 15 replicates (each sample analyzed 5 times in a day, for 3 consecutive days), as well as for the absolute and relative retention time and area values. Since the retention time was shown to be highly sensitive to the changes in the mobile-phase composition, each day a new mobile phase was freshly prepared by a different analyst. The method repeatability in terms of retention time and area was very high, exhibiting %RSD values ≤1.0 for both absolute and relative values. The intermediate precision showed a somewhat higher variation in retention time, although this was still below approximately 3% RSD. The changes in the mobile phase slightly affected the absolute retention times for the early eluting peaks, whereas the resolution for the later eluting peaks was observable as decreasing tendencies of absolute RT %RSD and increasing relative RT %RSD, respectively. The intermediate precision for the peak area was below 2% RSD for relative values, while for the absolute values, the maximum was up to approximately 6% RSD. The PMP–sugar derivatives in aqueous media can be decomposed at elevated temperatures, so refrigeration or drying is advised for prolonged storage [5]. In our study, the samples (standards mixture) were shown to be stable during 3 days of analyses, while being continuously tempered at 5 °C. However, for the highest precision, the use of the internal standard is mandatory in order to account for eventual losses.

The method recovery was evaluated by spiking a known sample with a known amount of sugar standard at three spiked concentrations. The results are presented in Table 3.

As a known sample, cocoa bean husk (CBH) was used, since it contained all monosaccharides (except ribose, used as internal standard) included in the development and optimization of the method in sufficient contents. At each spiked concentration level, the higher concentrations (100, 200 and 300 µg/mL) referred to glucose and galacturonic acid, as the most represented components in the sample, while the lower concentrations (25, 50, and 75 µg/mL) referred to the rest of sugars. The recovery rates for the lowest, middle and highest spiked concentration levels were approximately 95–106% with RSD ≤ 2.5%, 101–109% with RSD ≤ 2.7% and 101–110% with RSD ≤ 1%, respectively. At each level, the recovery was satisfactory, exhibiting an average error range of 5–10%. The obtained results correspond to previously published recovery data for similar derivatization protocols of monosaccharides using PMP [16,17].

### 3.3. Comparative Analysis of the Monomeric Composition of Cell Wall Polysaccharides of Selected Agro-Industrial Wastes

The developed HPLC-DAD method was used to determine the monomeric composition of cell wall polysaccharides of selected agro-industrial wastes, namely sugar beet pulp (SBP), walnut shell (WS), cocoa bean husk (CBH), onion peel (OP) and pea pod (PP). The results were compared to well-established and broadly used methods for monomeric profile determination, namely the alditol acetate method (GC-FID) for neutral sugars and the *m*-hydroxydiphenyl (MHDP) method for acidic sugars (Table 4). Both methods were chosen as references due to their long tradition of use and modifications, which have resulted in high efficiency. From the results of the HPLC analysis, it can be seen that glucose was the main component of the cell wall polysaccharides of the studied materials, probably referring to the cellulose content. Glucose was represented with approximately 22–25% dmb in the materials, except for CBH, where it comprised 16% dmb. Along with glucose, in SBP, CBH and OP, a significant content of GalA was found, in the range of approximately 8–20% dmb, indicating the abundance of pectins. In SBP, this was accompanied by a high content of arabinose (approximately 18% dmb). The results for the monomeric composition of SBP correspond to previous reports [39,40]. The GalA content determined by HPLC in CBH was lower than previously reported, at approximately 13.5% [41].

On the other hand, significant contents of xylose were found in WS and PP, along with glucose, at approximately 13% dmb (PP) to 20% dmb (WS), indicating the notable contents of xylan polymers. The determined xylose and hemicellulose contents in WS were in line with previous research [42,43], while for PP somewhat lower values were determined than in previous studies [44], which might be ascribed to the differences between the original samples.

In comparison to the reference GC and MHDP methods, a high correlation was found, especially regarding the neutral sugar content (Table 4). The biggest differences were found for acidic sugar content, GalA and GlcA, while higher values were obtained with the MHDP method. This can be explained by differences in the specificity of the applied methods. Although the specificity of the MHDP reagent for uronic acids is high, resulting in the development of pink-red derivatives, the interference of neutral sugars can be notable, arising from the formation of brown by-products from the reaction with strong mineral acids [37]. This can be especially true if the content of uronic acids is relatively low. From Table 4, it can be observed that samples with a high content of GalA, such as OP and SBP, showed less differences in the results than those having a low content of GalA, such as WS. Although the potential interference of neutral sugars was accounted for by evaluating the method’s responses towards xylose and glucose, the total interference of all sample components was difficult to estimate, which could have resulted in the overestimation of the GalA content. Another major difference can be observed regarding the content of GlcA. With the HPLC method, GlcA was found in WS, CBH and PP, while with the MHDP method was only found in in CBH. GlcA in the MHDP method was evaluated by subtracting the contents of total uronic acids and GalA. Regarding the possible interferences in the accurate determination of GalA (as well as total uronic acids), the content of GlcA might have been underestimated using the MHDP method, especially since it was found at low levels in the evaluated samples. In HPLC the acidic sugars were identified as separate peaks on chromatograms, meaning the HPLC method can be considered the preferred method for their quantification, while having the advantage of allowing the simultaneous analysis of neutral and acidic sugars in the same run, thereby saving time and chemicals. However, in a study by Wang et al. [15], it was shown that different types of sugar molecules require different derivatization (PMP) conditions to reach the maximum derivative yield; glucuronic acid reached maximum yield at a somewhat higher temperature and longer reaction time (approximately 3 °C and 20 min, respectively) in comparison to glucose. It was hypothesized that the presence and arrangement of different functional groups in the molecules resulted in molecular-level local changes in pH that can accelerate or hinder the reaction with PMP. Therefore, it cannot be excluded that applying only one set of reaction conditions for PMP derivatization, such as in the present study, may result in a slight underestimation of uronic acids.

## 4. Conclusions

Broadly available C18 columns are commonly used for the separation of PMP–sugar derivatives, although slight changes in the chemistry and preparation technology of these columns from different manufacturers complicate the transferability of established chromatographic conditions. The chromatographic separation of PMP–sugar derivatives is highly dependent on mobile-phase pH, as well as the content (and type) of the organic modifier.

In the present study, the higher pH values of the buffered component of the mobile phase and higher content of the organic component (acetonitrile) resulted in faster elution of PMP–sugar derivatives, affecting their resolution. Using the Zorbax Extend C18 column, the baseline separation of 8 neutral (mannose, ribose, rhamnose, glucose, galactose, xylose, arabinose and fucose) and 2 acidic sugars (glucuronic and galacturonic acids) was achieved by maintaining the buffered component of the mobile phase at pH 8.0 and the acetonitrile gradient at 12–17%. The established method showed high linearity, as well as repeatability and intermediate precision, and a satisfactory recovery error of 5–10% RSD. The limit of detection for analyzed sugars was generally lower than 6 µg/mL, whereas the highest limit of quantification was approximately 19 µg/mL, with both values being determined for glucuronic acid. The developed method was used to analyze 5 selected agro-industrial wastes (sugar beet pulp, walnut shell, cocoa bean husk, onion peel and pea pod) and gave results that were highly in line with the reference alditol acetates (GC) method for neutral sugars. In the uronic acid analysis, the HPLC and reference MHDP methods differed, with HPLC being the more preferred method. PMP derivatization combined with the developed HPLC method was shown to be simple, reliable and highly applicable in the evaluation of the monomeric compositions of polysaccharides from agro-industrial wastes as a primary analytical step in the process of their recovery and reutilization in various added-value products.

## Figures and Tables

**Table 1:** Linearity parameters, limit of detection (LOD) and limit of quantification (LOQ) of the developed HPLC-DAD method for the analysis of PMP–sugar derivatives.

Monosaccharide (PMP–Sugar Derivative)	Linear Equation	R^2^	Concentration Range (µg/mL)	LOD	LOQ
				(µg/mL)	
Mannose	y = 0.9037x + 0.0031	0.9999	10–400 (0.025–1) *	1.65	5.01
Rhamnose	y = 0.8679x + 0.0005	0.9999	10–400 (0.025–1) *	1.17	3.55
Glucuronic acid	y = 0.8068x − 0.0015	0.9996	10–400 (0.025–1) *	6.05	18.32
Galacturonic acid	y = 0.8275x − 0.0009	0.9996	10–400 (0.025–1) *	3.04	9.21
Glucose	y = 0.8535x − 0.0031	0.9998	10–400 (0.025–1) *	3.79	11.47
Xylose	y = 1.0185x − 0.0029	0.9998	10–400 (0.025–1) *	2.78	8.43
Galactose	y = 0.9861x − 0.0065	0.9998	10–400 (0.025–1) *	4.52	13.71
Arabinose	y = 1.1865x − 0.0049	0.9998	10–400 (0.025–1) *	3.85	11.67
Fucose	y = 0.873x − 0.0095	0.9995	10–400 (0.025–1) *	4.83	14.64

* Relative concentration range in relation to ribose, used as the internal standard.

**Table 2:** The precision parameters for the developed HPLC-DAD method for the analysis of PMP–sugar derivatives (RT—retention time; A—area).

		Method Precision (%RSD)			
		Repeatability (Intraday Precision)		Intermediate Precision (Interday Precision)	
		RT	A	RT	A
Mannose	Absolute	0.06–0.22	0.09–0.36	2.72–2.91	2.72–3.10
	Relative	0.01–0.03	0.02–0.32	0.11–0.13	0.18–1.63
Ribose	Absolute	0.07–0.21	0.07–0.45	2.84–3.02	3.02–4.26
	Relative	-	-	-	-
Rhamnose	Absolute	0.09–0.36	0.09–0.55	2.58–2.76	3.33–3.81
	Relative	0.01–0.03	0.09–0.55	0.26–0.26	0.34–0.97
Glucuronic acid	Absolute	0.09–0.31	0.04–0.50	0.69–0.89	3.50–4.04
	Relative	0.02–0.22	0.02–0.44	2.10–2.17	0.32–0.66
Galacturonic acid	Absolute	0.08–0.43	0.11–1.05	0.30–0.45	2.66–4.70
	Relative	0.02–0.31	0.08–0.88	2.54–2.60	0.45–1.04
Glucose	Absolute	0.08–0.19	0.06–1.32	0.85–1.00	3.23–4.54
	Relative	0.02–0.25	0.08–0.93	1.97–2.01	0.58–0.68
Xylose	Absolute	0.07–0.22	0.06–0.73	0.66–0.80	2.57–4.73
	Relative	0.02–0.26	0.04–0.32	2.16–2.22	0.48–0.68
Galactose	Absolute	0.07–0.15	0.02–1.15	0.38–0.51	1.60–5.82
	Relative	0.02–0.26	0.04–0.72	2.45–2.50	1.23–1.65
Arabinose	Absolute	0.07–0.15	0.04–1.00	0.35–0.47	2.84–5.39
	Relative	0.03–0.25	0.14–0.59	2.49–2.54	0.79–1.25
Fucose	Absolute	0.06–0.16	0.03–1.27	0.35–0.48	2.58–6.09
	Relative	0.03–0.25	0.14–0.85	2.48–2.53	0.50–1.91

**Table 3:** Recovery results of the developed HPLC-DAD method for the analysis of PMP–sugar derivatives, evaluated at three spiked concentrations of sugar standards.

Monosaccharide (PMP–Sugar Derivative	Recovery (%) at 25/100 µg/mL	RSD(%)	Recovery (%) at 50/200 µg/mL	RSD(%)	Recovery (%) at 75/300 µg/mL	RSD(%)
Mannose	102.06	1.13	106.44	0.24	104.95	0.28
Rhamnose	101.66	1.29	107.40	0.25	109.61	0.43
Glucuronic acid	99.15	0.17	104.42	1.01	103.57	0.95
Galacturonic acid	103.99	0.89	107.64	0.70	107.34	0.76
Glucose	99.40	0.00	103.30	0.33	100.97	0.29
Xylose	105.51	1.09	107.15	0.08	104.19	0.29
Galactose	99.95	2.44	101.43	0.01	100.53	0.47
Arabinose	101.32	1.62	108.92	0.10	108.73	0.41
Fucose	94.76	1.72	104.23	2.69	103.56	0.94

**Table 4:** Monomeric profile of selected agro-industrial wastes determined using the developed HPLC-DAD method and GC (alditol acetate) method in combination with the colorimetric (MHDP) method for acidic sugars (Man—mannose; Rha—rhamnose; GlcA—glucuronic acid; GalA—galacturonic acid; Glc—glucose; Xyl—xylose; Gal—galactose; Ara—arabinose; Fuc—fucose; SBP—sugar beet pulp; WS—walnut shell; CBH—cocoa bean husk; OP—onion peel; PP—pea pod; n.d.—not detected; dmb—dry matter basis). Values denoted with the same letter in superscript are not statistically different (*p* ≤ 0.05).

Monomer(% dmb)	SBP		WS		CBH		OP		PP	
	HPLC	GC + MHDP	HPLC	GC + MHDP	HPLC	GC + MHDP	HPLC	GC + MHDP	HPLC	GC + MHDP
Man	1.16 ± 0.02 ^a^	1.19 ± 0.04 ^a^	n.d.	n.d.	2.40 ± 0.00 ^a^	2.69 ± 0.06 ^a^	1.21 ± 0.05	1.33 ± 0.03	0.40 ± 0.00	0.64 ± 0.06
Rha	1.97 ± 0.06	1.26 ± 0.09	0.46 ± 0.03	0.41 ± 0.02	2.07 ± 0.02	1.33 ± 0.04	0.86 ± 0.05	0.58 ± 0.01	0.52 ± 0.00 ^a^	0.49 ± 0.03 ^a^
GlcA	n.d.	n.d.	2.10 ± 0.09	n.d.	1.23 ± 0.02 ^a^	1.33 ± 0.15 ^a^	n.d.	n.d.	0.58 ± 0.04	n.d.
GalA	15.29 ± 0.19	20.52 ± 0.54	1.96 ± 0.33	5.07 ± 0.14	7.95 ± 0.15	13.21 ± 0.26	19.74 ± 0.68	24.16 ± 0.56	4.24 ± 0.10	9.49 ± 0.40
Glc	21.85 ± 1.10 ^a^	20.85 ± 1.29 ^a^	25.09 ± 0.53 ^a^	22.97 ± 1.18 ^a^	16.13 ± 0.11	17.99 ± 0.58	25.30 ± 0.51	23.60 ± 0.64	19.41 ± 0.42 ^a^	20.92 ± 1.07 ^a^
Xyl	1.21 ± 0.06	1.57 ± 0.01	19.96 ± 0.73 ^a^	22.39 ± 0.68 ^a^	1.34 ± 0.02	1.80 ± 0.04	1.64 ± 0.03	2.06 ± 0.04	12.92 ± 0.05 ^a^	12.71 ± 0.86 ^a^
Gal	5.82 ± 0.35	5.24 ± 0.13	1.16 ± 0.23	1.08 ± 0.01	3.10 ± 0.00	3.54 ± 0.11	2.29 ± 0.19 ^a^	2.37 ± 0.12 ^a^	2.43 ± 0.33 ^a^	3.06 ± 0.19 ^a^
Ara	17.76 ± 0.78 ^a^	17.95 ± 0.58 ^a^	0.39 ± 0.07	0.54 ± 0.02	1.22 ± 0.00	1.62 ± 0.05	0.61 ± 0.03	0.76 ± 0.03	0.88 ± 0.17	1.39 ± 0.12
Fuc	0.15 ± 0.02 ^a^	0.18 ± 0.01 ^a^	n.d.	n.d.	0.16 ± 0.00	0.18 ± 0.01	0.24 ± 0.00 ^a^	0.25 ± 0.01 ^a^	n.d.	0.13 ± 0.01
TOTAL	65.22 ± 2.15	68.76 ± 1.64	52.76 ± 4.21	52.76 ± 1.73	35.59 ± 0.02	43.71 ± 0.96	51.76 ± 0.58	55.10 ± 0.85	40.73 ± 0.15	48.82 ± 1.68

## Data Availability

The data presented in this study are available on request from the corresponding author.
